# Distribution Characteristics and Risk Assessment of 57 Pesticides in Farmland Soil and the Surrounding Water

**DOI:** 10.3390/toxics12010085

**Published:** 2024-01-18

**Authors:** Weiqing Wang, Donghong Wang, Quanzhen Liu, Lihua Lin, Yongchang Xie, Chuan Du

**Affiliations:** 1 National Engineering Research Center of Industrial Wastewater Detoxication and Resource Recovery, Research Center for Eco-Environmental Sciences, Chinese Academy of Sciences, Beijing 100085, China; wqwang_st@rcees.ac.cn (W.W.);; 2 University of Chinese Academy of Sciences, Beijing 100049, China

**Keywords:** pesticides, Xingkai Lake, distribution, ecological risk assessment, metabolites

## Abstract

To investigate the effect of pesticide use on surface water, the concentration and distribution characteristics of 57 pesticides and 3 degradation products were analyzed in the farmland soil and surface water in the Xingkai Lake area, including water from paddy fields, drainages and the Xingkai Lake, in Heilongjiang Province, China. Forty-three pesticides and three degradation products were detected in farmland soil. In dry field (corn and soybean field) soil, the main detected pesticides were atrazine and acetochlor with mean concentrations of 26.09 ng·g^−1^ and 49.08 ng·g^−1^, respectively. In paddy field soil, oxadiazon, mefenacet and chlorpyrifos were the main detected pesticides with mean concentrations of 14.32 ng·g^−1^, 78.60 ng·g^−1^ and 20.03 ng·g^−1^, respectively. In the surrounding water, including water from paddy fields, drainages and Xingkai Lake, the total concentrations of contaminants detected in the water samples ranged from 71.19 ng·L^−1^ to 10,145.76 ng·L^−1^. Of the three sampling periods, the mean concentration of contaminants in the water exhibited its peak during the vegetative period. In the analysis of the drainage water, the primary pesticides detected were atrazine, acetochlor and buprofezin with mean concentrations of 354.83 ng·L^−1^, 109.09 ng·L^−1^ and 254.56 ng·L^−1^, respectively. Atrazine, simetryn, buprofezin and isoprothiolane were the main pesticides detected in Xingkai Lake water, with the mean concentrations of 222.35 ng·L^−1^, 112.76 ng·L^−1^, 301.87 ng·L^−1^ and 138.02 ng·L^−1^, respectively. The concentrations of contaminants could be correlated with drainage, Da Xingkai Lake and Xiao Xingkai Lake water (ρ > 0.8) suggested that the source of these contaminants in drainage and Xingkai Lake water could be the same. The maximum potentially affected fraction (PAF) values of atrazine, chlorpyrifos and prometryn were higher than 5% in Xingkai Lake water, resulting in high ecological risks.

## 1. Introduction

Pesticides are chemical agents used in agriculture to control pests and diseases and regulate plant growth [1]. Pesticides currently in use include organophosphorus, amides, carbamates, azole and so on. As the research on pesticides continues to develop, people have found that organophosphorus insecticides can cause neurotoxic effects by inhibiting acetylcholinesterase. This has adverse effects on the human visual system [2] and even affects the normal development of the fetus [3]. Atrazine in triazine pesticides has endocrine disrupting effects [4] and is classified as an endocrine disruptor by the European Union. Acetochlor in amide herbicides can affect the expression of the thyroid hormone gene in fish [5], while butachlor has toxic and endocrine-disrupting effects on zebrafish embryos [6]. Unfortunately, in China, the annual use of atrazine totals more than 1000 tons [7], and the annual use of alachlor, acetochlor and butachlor, as the three main amide herbicides, totals more than 100,000 tons [8]. Less than 0.1% has an active effect on disease control [9], with the rest forming a residue in the environment.

Such a high concentration of pesticides forms residues in the soil, affects the soil environment and pollutes the water environment through surface runoff, soil leaching [10] and so on, affecting the aquatic organisms. The pollution of surrounding surface water caused by pesticide use is constantly being reported. A total of 83 pesticides have been detected, and imidacloprid was shown to pose a high risk to sensitive ecological species in the northwest of Taihu Lake [11]; in Poyang Lake, neonicotinoid insecticides and organochlorine pesticides were detected [12,13]. The maximum concentration of pesticides detected in Lake Biwa, Japan, was 0.4 μg·L^−1^ [14]. The surface water of the northern Indo-Gangetic alluvial plains was affected by agricultural activities, and the concentration of organochlorine pesticides in the surface water was 2.63 μg·L^−1^~3.72 μg·L^−1^ [15]. They are all the important local surface water resources, but due to the development of agriculture in the surrounding areas, many kinds of pesticides remain in the surface water. However, Xingkai Lake, as the largest freshwater lake in Northeastern Asia [16] with a large amount of farmland surrounding it, has been rarely reported on in relation to pesticide residues.

Xingkai Lake, located in the southeast of Heilongjiang Province, China, is composed of Da Xingkai Lake and Xiao Xingkai Lake and is a located on the boundary between China and Russia. Xingkai Lake is located in a significant local grain production zone, characterized by minimal industrial pollution and predominantly agricultural activities [17,18]. The pollution in the lake is mainly from farmland. According to *The Ecological and Environmental Status Bulletin* of the Chinese government [19], Xingkai Lake is a Class V surface waterbody that is moderately polluted and has a light eutrophic status. In the past, research on the water environment of Xingkai Lake mostly focused on the water quality [20,21]. Although residues of acetochlor, butachlor and organochlorine pesticides have been reported in the farmland soil and surface water of Xingkai Lake [22,23], many other pesticides have not been reported. At the same time, Xingkai Lake is rich in fish and has a developed fishery industry. The effect of the use of agricultural pesticides on the aquatic ecological environment of Xingkai Lake is still unclear.

Therefore, the objective of this study was to explore the current levels of pesticide residues in the Xingkai Lake area and reveal the impact of the use of agricultural pesticides on the water environment of Xingkai Lake. We investigated the concentrations and distribution characteristics of 57 pesticides in the farmland soil and water around Xingkai Lake, including paddy field water, drainage water and Xingkai Lake water, using gas chromatography-mass spectrometry (GC-MS).

## 2. Materials and Methods

### 2.1. Materials and Reagents

The 57 pesticides and 3 degradation products that were used in this study were divided into six categories according to chemical group as shown in Table 1, including amides/anilines, azoles, carbamates, heterocyclic, organophosphates and triazines. The names, CAS numbers and companies from which the 57 pesticides and 3 degradation products were purchased are listed in Table S2. The recovery indicators phenanthrene-d10 and atrazine-d5 were purchased from Accustandard, New Haven, CT, USA. All of the solvents used in this study, including methanol, n-hexane (HEX) and dichloromethane (DCM), were of high-performance liquid chromatography (HPLC) grade (Thermo Fisher Scientific, Waltham, MA, USA). Glass fiber filters with a 0.7 μm aperture were purchased from Millipore, Billerica, MA, USA; anhydrous sodium sulfate was purchased from Sinopharm Chemical Reagent Co., Ltd., Shanghai, China; ultrapure water was prepared using a RephiLe-J24617 system (PURIST PRO, Lefeng Biotechnology Co., Ltd., Shanghai, China); the C18 (6 cc 500 mg) solid phase extraction (SPE) columns and HLB (6 cc 500 mg) SPE columns were purchased from Waters, Milford, MA, USA; celite was purchased from J&K Scientific, Beijing, China; and Florisil (6 mL, 1 g) SPE columns were purchased from Supelco, Merck, Darmstadt, Germany.

### 2.2. Study Sites and Sampling

**Figure 1:**
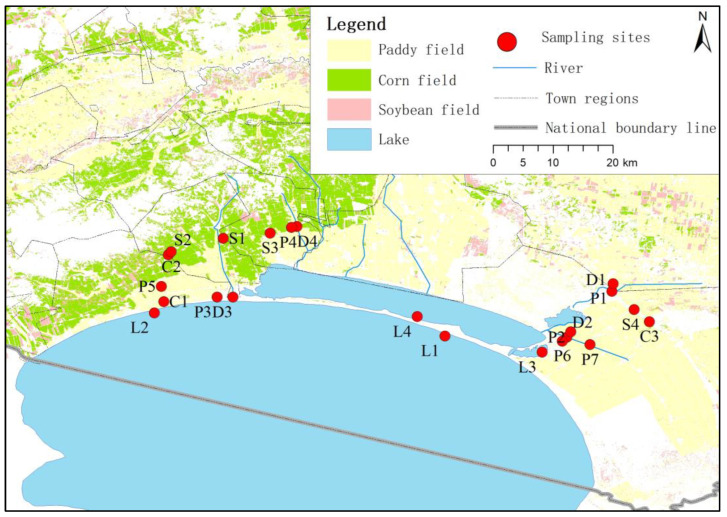
Distribution of sampling sites of farmland soil and surface water.

As shown in Figure 1, samples were collected at the 22 sampling sites during May 2022 (sowing period), July 2022 (vegetative period) and September 2022 (maturity period), including surface water samples and farmland soil samples. Detailed information regarding the sampling sites is shown in Table S1.

The farmland soil samples were collected at 14 sampling sites, including corn fields (C1~C3), paddy fields (P1~P7) and soybean fields (S1~S4). The soil samples were collected using a five-point sampling method, and the four surrounding sampling points were 20 cm away from the center point. Soil samples were taken 10 cm below the crop root with a stainless steel spoon, and about 100 g was collected at each point. The sampling points were arranged to avoid farmland boundaries, and the collected samples were preserved at a low temperature of −20 °C.

The 26 surface water samples were collected at 12 sampling sites, including paddy field water, drainage water and Xingkai Lake water. The sampling points for paddy field water were designated P1 through to P4, while the drainage water was sampled at D1 through to D4. Additionally, the sampling points for Da Xingkai Lake were identified as L1 and L2, whereas those for Xiao Xingkai Lake were noted as L3 and L4. A total of 4 L of water was collected from each sampling point with a stainless steel vessel, and the sampling points were located 50 cm away from the shore, with a collection depth of 20~30 cm below the surface of the water. Obvious pollution sources were avoided during sampling. The samples were subsequently gathered in brown glass bottles and transported to a storage location at a temperature of −4 °C. Following this, we underwent pretreatment within a 48 h period.

### 2.3. Sample Pretreatment

#### 2.3.1. Soil Sample Pretreatment

The soil was sieved to a particle size of less than 2 mm after being freeze-dried using Freeze Dry Systems (Freezone 4.5, Labconco, TX, USA). Accelerated solvent extraction (ASE) [24,25] was carried out with an ASE 350 extractor (Dionex, Sunnyvale, CA, USA). The extraction program for soil samples was as follows: 5 g of soil was mixed with 2 g of celite in a 34 mL stainless steel vessel and 200 ng each of the recovery indicators phenanthrene-d10 and atrazine-d5 was added; then, acetone: HEX (1:1, *v*/*v*) was used as the extraction solvent at 100 °C under a pressure of 1500 psi with 5 min of heating and 5 min of static extraction, which was carried out in two cycles; and the extraction cell was flushed with 60% of the cell volume of the solvent and purged with nitrogen for 60 s [26]. The extract was dehydrated using anhydrous sodium sulfate and then concentrated to 2 mL using a rotary evaporator (Heidolph, Schwabach, Bavaria, Germany). After the extraction procedures, the extracts were transferred to a Florisil column that was used to clean up interfering substances. The columns were activated with 10 mL of each of DCM and HEX before use. The target components were eluted with 15 mL of leachate (DCM: HEX = 1:1, *v*/*v*) [26,27]. The elute was concentrated to dryness with a gentle nitrogen flow, and then the solvent was replaced with HEX, setting the volume to 0.5 mL, and maintained at −20 °C for subsequent instrumental analysis.

#### 2.3.2. Water Sample Pretreatment

Particles were first filtered from the water samples using 0.7 μm glass fiber filters [28]. Next, 2 L of each filtered water sample was taken in a brown glass bottle, and then 100 ng of the recovery indicator phenanthrene-d10 was added for solid phase extraction. The solid phase extraction method was used for the pretreatment of the water sample, and the SPE columns C18 and HLB were selected in tandem. The C18 and HLB columns were activated with 10 mL of each of DCM, methanol and ultrapure water. The C18 and HLB SPE columns were enriched in series using negative pressure. The components were eluted with 10 mL of DCM. The elute was dehydrated using anhydrous sodium sulfate and concentrated to dryness with a gentle nitrogen flow, and then HEX was added to replace the solvent, setting the volume to 0.5 mL. The solution was maintained at −20 °C for subsequent instrumental analysis [29,30].

### 2.4. Instrumental Determination

Gas chromatography-mass spectrometry (GC-MS) was used to analyze the 57 pesticides and 3 degradation products (6890N-5975B, MSD, Agilent Technologies Inc., Santa Clara, CA, USA). The capillary column used was a DB-5MS (30 m × 0.25 mm × 0.25 μm, J&W Scientific, Folsom, CA, USA). Nitrogen was used as the carrier with a constant flow velocity of 1.0 mL·min^−1^. The injection volume was 1.0 μL with a splitless inlet. Qualitative analysis was performed in scan mode, and quantitative analysis was performed in selected ion mode (SIM) [29,30]. Chromatographic quantitative ion (*m*/*z*) and retention time are shown in Table S2.

### 2.5. Quality Assurance and Quality Control

The methods were verified by measuring pesticide recovery, the limit of detection (LOD), the limit of quantitation (LOQ) and the regression coefficient (R^2^) before sample analysis. The validation of water and soil sample pretreatment methods was carried out using matrix recovery tests. Ultrapure water was used as the matrix for the water sample method validation experiments, and the actual soil samples were used as the matrix for the soil pretreatment experiments. The mass of the target compound added to the matrix was 150 ng, and the pretreatment process was the same as that of the actual samples. The validity of the method for water and soil samples was confirmed separately. A total of three groups of matrix blank parallel experiments and five groups of matrix addition target object parallel experiments were set up to calculate the target recovery rate. The LOD and LOQ of each pesticide and degradation product were calculated according to the concentrations corresponding to a signal/noise (S/N) of three and ten for pure standard solutions. The recovery rate ranged from 74.8% to 107.8% in water and 60.9% to 109.3% in soil. The recovery rates of the recovery indicators phenanthrene-d10 and atrazine-d5 added during the pretreatment were 86.0%~119.1% and 84.3%~118.8%. The established GC-MS method had a wide linear range and a good correlation coefficient (R^2^ > 0.99). The detailed information is shown in Table S2.

### 2.6. Ecological Risk Assessment

The species sensitivity distribution (SSD) method was used to assess the ecological risk of the pesticides detected in Xingkai Lake; it combines the toxicity data from multiple single species to predict the concentrations affecting a given number of species in a community [31,32]. It is widely used to evaluate the ecological risk of a pollutant or pollutants to organisms [33]. This study was carried out according to the following steps: (1) toxicity data acquisition; (2) SSD curve fitting; (3) the calculation of the potential affected fraction (PAF); and (4) the ecological risk assessment of single compounds.

The biotoxicity data used in SSD were obtained from the US EPA ECOTOX database (https://cfpub.epa.gov/ecotox/ (accessed on 28 November 2023)), Chinese academic literature (CNKI, http://www.cnki.net/ (accessed on 28 November 2023)) and English academic literature (http://www.sciencedirect.com/ (accessed on 28 November 2023)), and acute toxicity data were used to construct an SSD curve and the lethal concentration (LC_50_) or median effective concentration (EC_50_) as the toxicity endpoint. The exposure medium was fresh water and the exposure time was no more than 4 days.

Although many methods may be used to carry out the cumulative distribution function [34,35], the log-logistic distribution was used since it often fits toxicity data well [31,36].

(1) $$y=\frac{a}{1+e^{-k(x-x_{c})}}$$

where y is the proportion of species affected, which is defined by ranking and numbering toxicity data from smallest to largest and labelling them 1 to n, and then calculating $\frac{n}{1+n}$; x is the logarithmic value of the toxicity data; a is the amplitude; $x_{c}$ is the parameter of location; and *k* is the parameter of the slope of the curve.

The potentially affected fraction (PAF) of species was calculated for each exposure concentration of the monitored pesticides to evaluate the ecological risk. When the PAF is less than 5%, the ecological risk is low or not significant. However, when the calculated PAF equals or exceeds 5% of the species, the ecological risk escalates significantly [37]. To calculate the y, that is, the PAF value, x is taken as the concentration value of the component and brought back to Formula (1).

## 3. Results

### 3.1. Residual Characteristics of Contaminants in Farmland Soil

During the sampling procedure, pesticide bottles containing bentazon were discovered discarded adjacent to the field. Due to the limited response of GC-MS in directly detecting bentazon, an analysis of degraded bentazon methyl was conducted. In this analysis, the detection rate of atrazine was observed to be significantly higher. The degradation products of atrazine, which are atrazine-desisopropyl and desethylatrazine, are highly toxic to aquatic organisms [38]; therefore, we also analyzed atrazine-desisopropyl and desethylatrazine.

**Figure 2:**
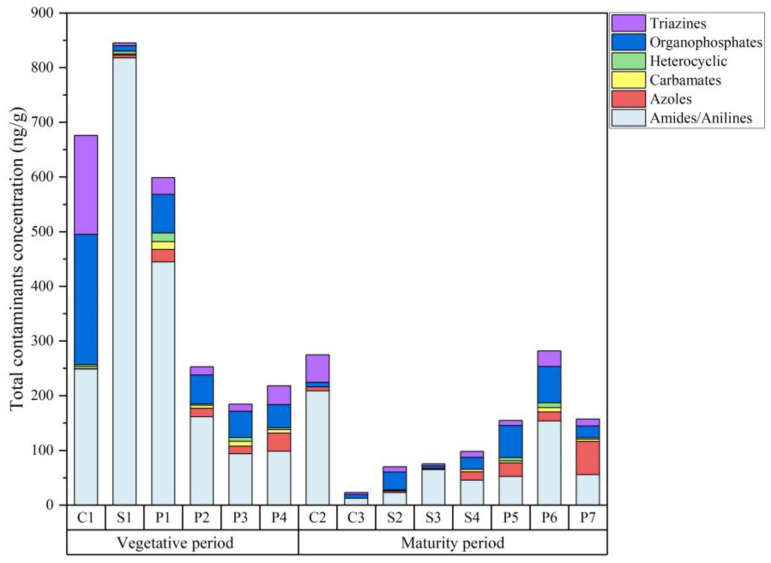
Total concentrations of contaminants and the types of chemical constituents in farmland soil at different sampling points.

A total of 43 pesticides and 3 degradation products were detected in farmland soil, of which amides and anilines were the main component. The total concentrations of contaminants and the types of chemical constituents in the soil samples are shown in Figure 2. In the vegetative period, 44 contaminants were detected with total concentrations of 189.02 ng·g^−1^~845.27 ng·g^−1^, and 41 contaminants were detected with total concentrations of 22.99 ng·g^−1^~281.94 ng·g^−1^ in the maturity period. The concentrations of pesticides and degradation residues in the vegetative period were higher than those in the maturity period, primarily because the vegetative period is the primary application period for pesticides.

**Figure 3:**
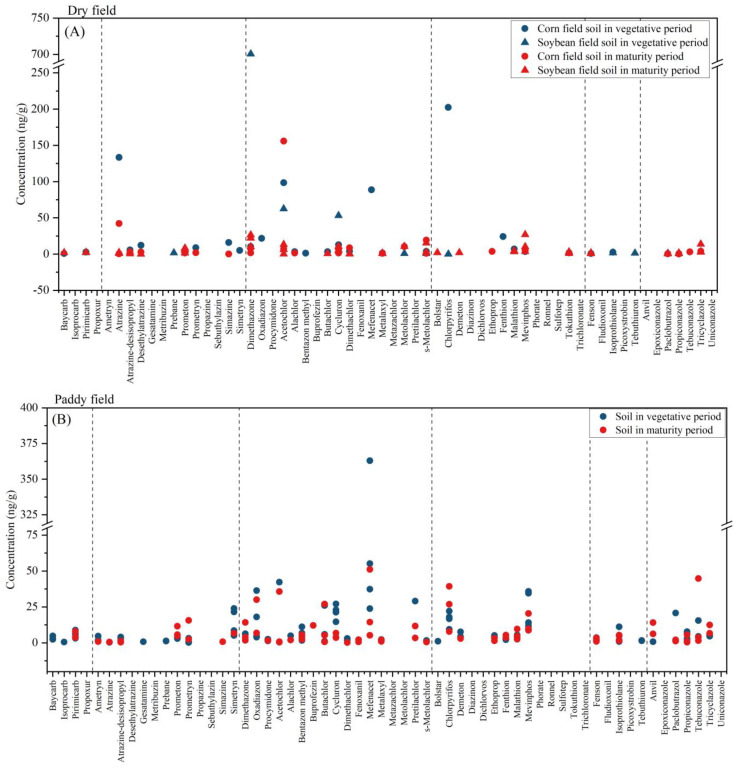
Concentrations of the 60 contaminants detected in dry field (**A**) and paddy field (**B**) soil.

In dry field (corn and soybean field) soil (Figure 3A), 34 pesticides and 3 degradation products were detected, and the mean concentration of contaminants was 760.62 ng·g^−1^ in the vegetative period and 108.25 ng·g^−1^ in the maturity period. The main detected pesticides were atrazine and acetochlor. Atrazine and acetochlor are applied in the vegetative season. The maximum concentrations of these two herbicides were, respectively, 133.53 ng·g^−1^ and 155.75 ng·g^−1^. Atrazine was regularly reported in dry field soil, and its mean concentration was 26.09 ng·g^−1^ in the dry fields in the Xingkai Lake area. Compared with other studies, this level was notably higher than that found in the agricultural soils of Liaoning (18.38 ng·g^−1^) and in riparian soils in the Songhua River Basin (11.28 ng·g^−1^) [39,40]. Yu’s research [22] conducted in the Xingkai Lake area revealed an acetochlor detection rate exceeding 80%, with a maximum recorded concentration of 117.1 ng·g^−1^. This finding aligns with the results of our study.

In paddy field soil (Figure 3B), 40 pesticides and 2 degradation products were detected. The elevated levels of pesticides detected in paddy fields, compared to dry fields, may be attributed to the distribution of pesticides within the water and soil of these paddy fields. The mean concentration of contaminants was 314.87 ng·g^−1^ in the vegetative period and 198.01 ng·g^−1^ in the maturity period. The main detected pesticides were oxadiazon, mefenacet and chlorpyrifos. The data indicate that mefenacet was the predominant local herbicide in the paddy field, with a peak concentration of 363.00 ng·g^−1^ and an average concentration of 78.60 ng·g^−1^. The maximum observed concentration of butachlor was 27.03 ng·g^−1^, which was lower than the previously reported value of 140.55 ng·g^−1^ [22]. The highest concentration of organophosphates was 26.85 ng·g^−1^, which was lower than the maximum residues in the paddy fields of northern Thailand (58.6 ng·g^−1^) [41]. In paddy field soil, the fungicide detection rate was 50.6%, whereas in dry field soil, it was 22.1%. In paddy field soil, the fungicide with the maximum concentration was tebuconazole (44.82 ng·g^−1^). In contrast, the most prevalent fungicide in dry field soil was tricyclazole, with a concentration of 13.75 ng·g^−1^. The data suggest that the frequency and concentration of fungicides detected in paddy field soil surpass those found in dry field soil. This discrepancy may be attributed to the humid conditions prevalent in paddy fields, which render them more prone to mildew.

### 3.2. Residual Characteristics of Contaminants in Surrounding Water

**Figure 4:**
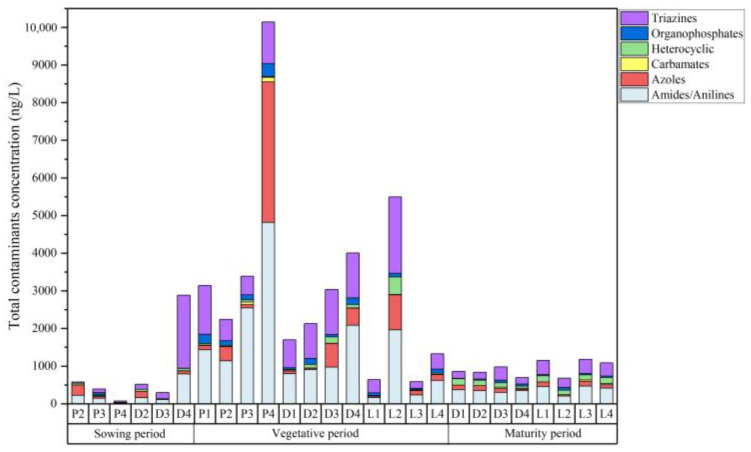
Total concentrations of contaminants and the types of chemical constituents in the surrounding water at different sampling points.

In the surrounding water samples, 48 pesticides and 3 degradation products were detected, of which 8 pesticides had a detection rate of 100%, namely atrazine, acetochlor, simetryn, prometryn, isoprothiolane, metolachlor, oxadiazon and fenoxanil. The total concentrations of contaminants and the types of chemical constituents in the water samples are shown in Figure 4. The total concentrations of the contaminants detected in the water samples ranged from 71.19 ng·L^−1^ to 2883.94 ng·L^−1^ in the sowing period, from 585.46 ng·L^−1^ to 10,145.76 ng·L^−1^ in the vegetative period and from 680.38 ng·L^−1^ to 1178.93 ng·L^−1^ in the maturity period. The concentration of residual contaminants in the vegetative period was higher than that in the sowing and maturity periods, which was consistent with increased pesticide use in the vegetative period.

**Figure 5:**
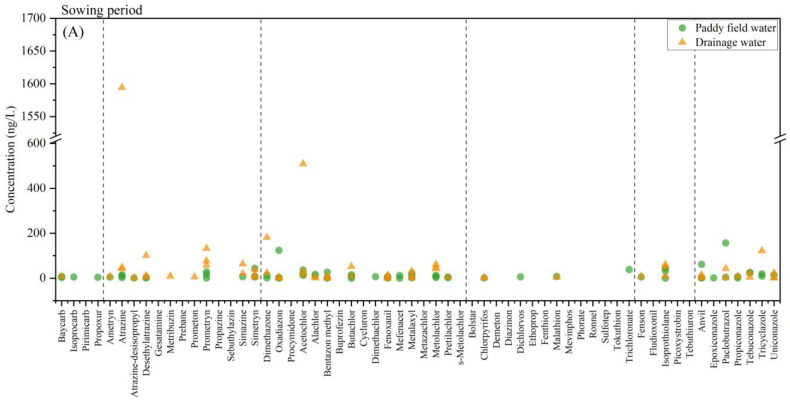

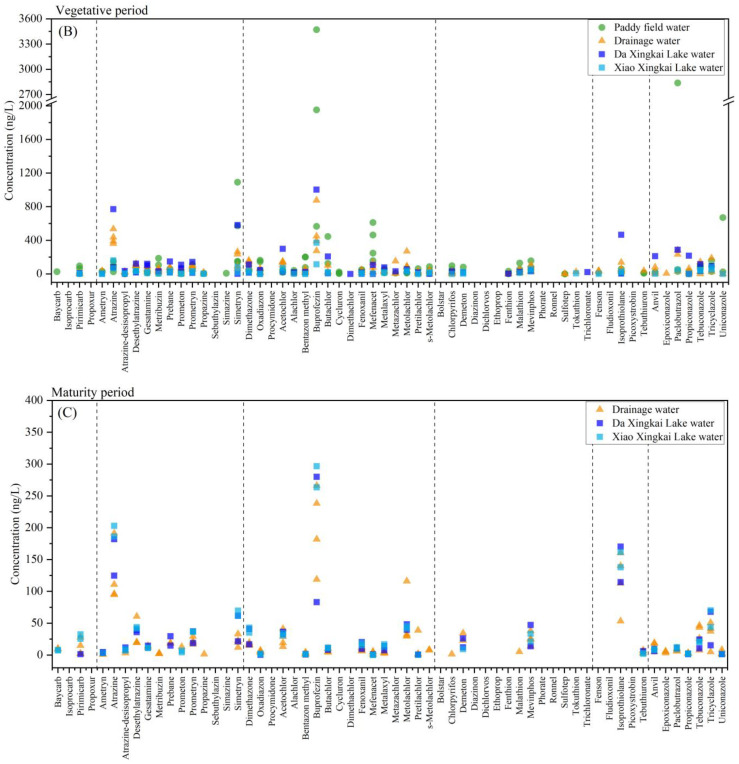
Concentrations of the 60 contaminants detected in the sowing (**A**), vegetative (**B**) and maturity (**C**) periods in surrounding water.

In the sowing period (Figure 5A), almost no pesticides were used. The mean total concentration of contaminants was 346.47 ng·L^−1^ in paddy field water and 1233.50 ng·L^−1^ in drainage water. Except for the D4 sampling points, the concentration of contaminants at other sites was minimal, which can be considered to be representative of the environmental background value. The maximum values of atrazine, prometryn, dimethazone and acetochlor were all detected in D4, and they totaled 1594.60 ng·L^−1^, 132.83 ng·L^−1^, 181.88 ng·L^−1^ and 509.60 ng·L^−1^. This may have been due to the fact that the D4 point was close to a corn field that was affected by the use of these substances or other human factors.

In the vegetative period (Figure 5B), the main contaminants were triazines, amides and azole, including seven pesticides: atrazine, simazine, acetochlor, buprofezin, butachlor, mefenacet and paclobutrazol. The mean concentrations across various samples were as follows: 4729.69 ng·L^−1^ in paddy field water, 2717.84 ng·L^−1^ in drainage water, 3067.80 ng·L^−1^ in water from Da Xingkai Lake and 955.91 ng·L^−1^ in water from Xiao Xingkai Lake. The highest concentration of contaminants was observed in paddy field water, a finding that can be attributed to the direct use of pesticides within these fields. According to the results of the study, buprofezin might be the main insecticide used in the Xingkai Lake area, with a mean concentration of 891.18 ng·L^−1^. Buprofezin is typically applied during the summer months, specifically from late July to early August. It is plausible that our sampling occurred during the vegetative period, coinciding with the use of buprofezin. This could have led to elevated residual concentrations in the paddy field water. The primary herbicides detected in the study were simazine, butachlor and mefenacet. The mean concentrations of these herbicides in water were 203.60 ng·L^−1^, 103.10 ng·L^−1^ and 114.43 ng·L^−1^, respectively. These herbicides were also highly prevalent in paddy field soil. During the vegetative period, the mean concentrations of atrazine and paclobutrazol in water were found to be 297.81 ng·L^−1^ and 375.18 ng·L^−1^, respectively.

In the maturity period (Figure 5C), the main contaminants were atrazine, buprofezin and isoprothiolane with mean concentrations of 148.76 ng·L^−1^, 216.19 ng·L^−1^ and 131.56 ng·L^−1^. The mean total concentrations were 841.74 ng·L^−1^ in drainage water, 914.53 ng·L^−1^ in Da Xingkai Lake water and 1133.68 ng·L^−1^ in Xiao Xingkai Lake water. To maintain pesticide residues in crop grains within safe limits, the use of pesticides is progressively halted during the early period prior to harvest. Consequently, the concentration of these residues diminishes during the maturity period compared with the vegetative period.

The use of pesticides on farmland has had a detrimental impact on Xingkai Lake, resulting in the detection of various contaminants in its water. In Da Xingkai Lake and Xiao Xingkai Lake water, 37 pesticides and 3 degradation products were detected. Atrazine, simetryn, buprofezin and isoprothiolane were the main detected pesticides. Atrazine is widespread in surface water in China; the concentration range was 66.80 ng·L^−1^~772.15 ng·L^−1^ in Xingkai Lake water, which was similar to the concentrations in the Songhua River and the Heilongjiang River of 844.9 ng·L^−1^ and 606.5 ng·L^−1^ [42]. The maximum concentration of simetryn was 581.80 ng·L^−1^ and the median concentration was 61.80 ng·L^−1^, while the maximum concentration of simetryn reported in surface water in Wuhan and the Liao-He River was only 10.7 ng·L^−1^ [43,44], which is lower than that in this study. The mean concentration of acetochlor was 70.88 ng·L^−1^, which is lower than that reported in the major growing areas in China (4.3 μg·L^−1^) [8]. Buprofezin was detected with a mean concentration of 301.87 ng·L^−1^, and its maximum concentration reached 1003.44 ng·L^−1^ in the vegetative period. In the Taige Canal basin, buprofezin concentrations ranged from 56 ng·L^−1^ to 390 ng·L^−1^, averaging at 218 ng·L^−1^ [45]. This finding aligns with the results obtained in this study. The contamination of isoprothiolane was notably high during the maturity period, with a mean concentration of 129.66 ng·L^−1^ in the vegetative period and an increase to 146.38 ng·L^−1^ in the maturity period. Wang also reported [11] a higher concentration of isoprothiolane (186 ng·L^−1^) in September, which is consistent with the findings of this research.

Xingkai Lake and Taihu Lake, both freshwater bodies, are significantly impacted by the use of pesticides on farmland. Upon comparing the pesticide levels in Xingkai Lake to those in Taihu Lake, it was observed that the concentration of butachlor was markedly higher than in Taihu Lake. The peak concentration of butachlor in Taihu Lake was recorded at 43 ng·L^−1^, with a mean concentration of 1.5 ng·L^−1^ [11]. In contrast, Xingkai Lake exhibited a maximum concentration of 209.22 ng·L^−1^ for butachlor, accompanied by a mean concentration of 36.58 ng·L^−1^. There were also significant differences between the residues of dichlorvos in Xingkai Lake and in Taihu Lake. Wang [11] reported that the highest average concentration of dichlorvos was measured at 4.2 ng·L^−1^ with a maximum value of 263 ng·L^−1^ in Taihu Lake; Zhou [46] reported that the mean value of dichlorvos in the Meiliangwan Bay of Taihu Lake was 51.6 ng·L^−1^; however, no dichlorvos was detected in Xingkai Lake during this study. These factors could be associated with the cultivated crops and the variations in pesticide usage between the northern and southern regions [8]. Interestingly, a lake in Japan had similar findings to Xingkai Lake. Lake Biwa, as the largest lake in Japan [41], is also heavily impacted by surrounding agricultural activities. In Japan, simetryn and isoprothiolane are also applied in paddy fields. In Lake Biwa, both simetryn and isoprothiolane had high detection rates with the maximum concentrations of 0.41 μg·L^−1^ and 0.15 μg·L^−1^ [41], while dichlorvos was also not detected in the lake. The results of this study were similar to those in Xingkai Lake, possibly because the pesticides used in these two areas are similar.

**Figure 6:**
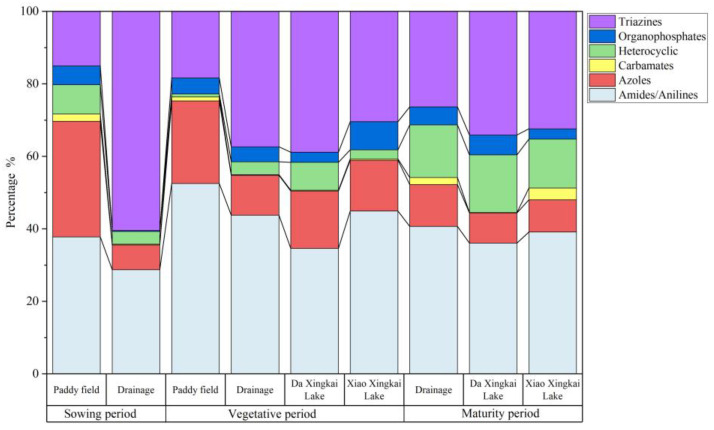
Proportion of contaminants with different types of chemical constituents in surrounding water.

The proportions of the different types of contaminants in the surrounding water in different periods are illustrated in Figure 6. The concentration of carbamate pesticides was found to be the lowest, potentially due to their high toxicity and thus their being gradually phased out [47]. Triazines and amides were the main detected pesticides in the surrounding water. During both the vegetative and maturity periods, the proportions of various structural contaminants were comparable in both drainage and Xingkai Lake water. The proportions of amide pesticides in drainage, Da Xingkai Lake and Xiao Xingkai Lake water during the maturity period were 40.7%, 39.1% and 36.1%, respectively. The distribution of heterocyclic pesticides exhibited similarity across drainage and Xingkai Lake water. We conducted a Spearman correlation analysis of the contaminants in the drainage and Xingkai Lake water using Origin 2019 as our analytical tool. The results of this correlation, represented by the correlation coefficient (ρ), are shown in Table 2. The results showed that the distribution characteristics of the contaminants could have a correlation in the drainage, Da Xingkai Lake and Xiao Xingkai Lake water (ρ > 0.8). Furthermore, the correlation was more pronounced during the maturity period compared to the vegetative period. This suggested that the source of these contaminants in the drainage and Xingkai Lake water could be the same. Similar findings were observed in Taihu Lake, where the cumulative concentration of pesticides detected in its northwest region mirrored that of the upstream river. This suggests that the catchment encompassed by the study area could potentially be the primary source of the pesticides in the northwest region of Taihu Lake [11].

### 3.3. Ecological Risk Assessment

**Figure 7:**
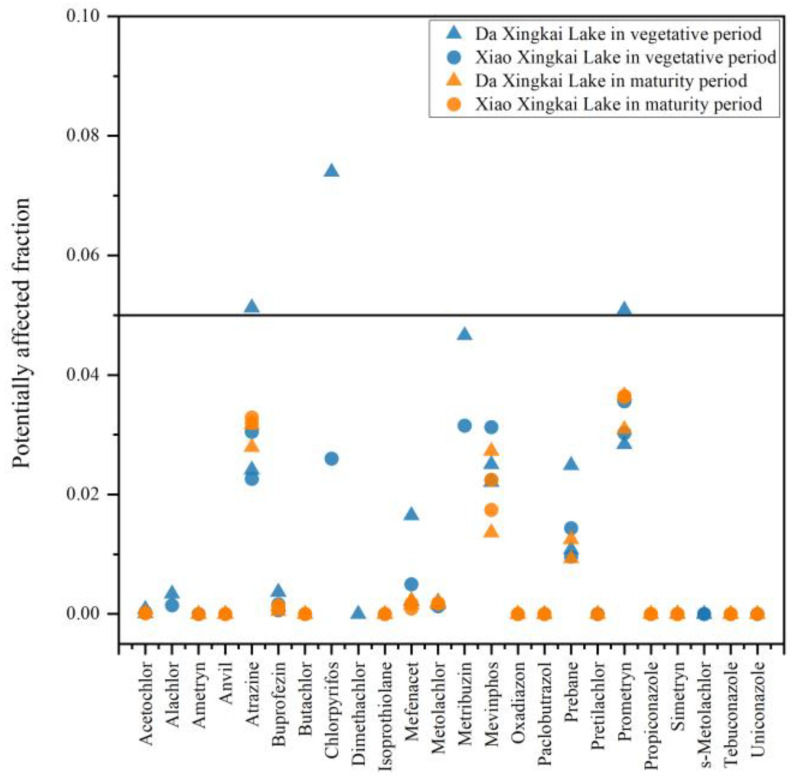
Potentially affected fraction of 24 pesticides in Xingkai Lake water.

To evaluate the possible toxic effects of contaminants on aquatic organisms, an ecological risk assessment was carried out on Da Xingkai Lake and Xiao Xingkai Lake water during different periods. This investigation scrutinized the ecological risks posed by 24 types of highly concentrated and frequently detected pesticides. The findings are presented in Figure 7. The summaries of the regression parameters and the regression coefficient of the SSD curve are shown in Figure S1 and Table S3. The PAF values in the vegetative period were higher than in the maturity period. The maximum PAF values of atrazine, chlorpyrifos and prometryn were higher than 5% in the vegetative period, totaling 5.13%, 7.40% and 5.09%, respectively, so atrazine, chlorpyrifos and prometryn could constitute significant ecological risks. In Taihu Lake, the maximum concentration of atrazine was 614 ng·L^−1^ [46]. When this maximum value was incorporated into our formula, it yielded a PAF value of 4.75%, which is nearly equivalent to 5%. This suggests potential risks to aquatic organisms. C.S. Qu [48] also reported atrazine as one of the greatest hazards to the species in Taihu Lake wetlands. The pesticide metribuzin exhibited a maximum PAF of 4.66%, which was proximate to the threshold of 5%, indicating that it warrants attention. In the maturity period, the PAF values of atrazine and prometryn were still higher than those of other contaminants. Although they did not surpass 5% (3.29% and 3.66%, respectively), these levels still pose a certain risk to the aquatic ecological environment. These results indicated that the harm of atrazine and prometryn to aquatic organisms may exist for a long time Xingkai Lake. To sum up, attention should be paid to the toxic effects of atrazine, chlorpyrifos and prometryn on aquatic organisms.

The aquatic life benchmarks for atrazine, chlorpyrifos and prometryn in freshwater vertebrates and invertebrates were retrieved from USEPA [49]. Detailed information is shown in Table 3. In Xingkai Lake water, the maximum concentrations of atrazine, chlorpyrifos and prometryn were 772.15 ng·L^−1^, 29.71 ng·L^−1^ and 144.00 ng·L^−1^. Both atrazine and prometryn concentrations were below the acute freshwater invertebrate and vertebrate benchmarks, but the chlorpyrifos concentration was above the acute freshwater invertebrate benchmark. This implied that the residual concentration of chlorpyrifos in Xingkai Lake water poses a risk to freshwater invertebrates. Atrazine and prometryn did not present a risk based on the EPA benchmarks, probably due to the fact that the benchmarks are pending an update.

**Figure 8:**
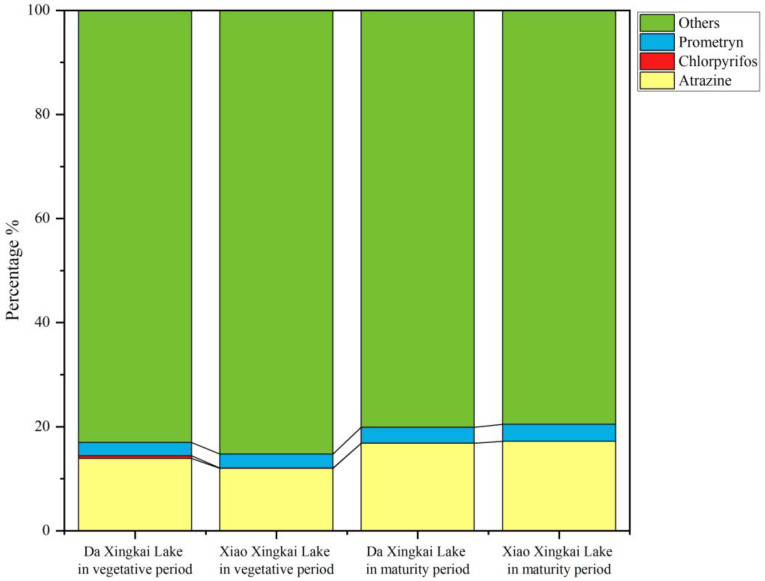
The proportion of high-risk pesticide concentrations in Xingkai Lake.

To better illustrate the characteristics of high-toxicity pesticide (atrazine, chlorpyrifos and prometryn) residues in Xingkai Lake water, we evaluated the proportion of high-toxicity pesticides to total pesticide concentrations. The results are shown in Figure 8. The proportion of high-toxicity pesticides to total pesticide concentrations ranged from 14.71% to 20.43%, with atrazine being the most dominant at 11.97% to 17.18% of the total concentrations. The highest ecological risk was for chlorpyrifos, but the proportion of the maximum concentrations was only 0.48%. Therefore, the residual pesticides in Xingkai Lake water are mainly low-toxicity pesticides.

## 4. Conclusions

In the farmland soil of the Xingkai Lake area, 43 pesticides and 3 degradation products were detected. In paddy field soil, 40 pesticides were detected, while in dry field soil, 34 pesticides were identified. The main pesticides were atrazine and acetochlor in dry field soil, and oxadiazon, mefenacet and chlorpyrifos in paddy field soil.

In Xingkai Lake water, atrazine, simetryn, buprofezin and isoprothiolane were the main pesticides among 37 pesticides detected. The pesticides and degradation products detected in Xingkai Lake water were detected in both paddy field water and drainage water. The distribution characteristics of contaminants in the drainage water, Da Xingkai Lake and Xiao Xingkai Lake water could have a correlation, and so the sources could be the same. In the vegetative period, atrazine, chlorpyrifos and prometryn could pose an environmental risk to aquatic organisms in Xingkai Lake water.

The use of pesticides on farmlands may cause pesticide residues in Xingkai Lake water. Over time, the aquatic environment of Xingkai Lake may be seriously affected and even cause species death, which needs to be noticed.

## Figures and Tables

**Table 1:** Classification of the 57 pesticides and 3 degradation products.

Subtype	Pesticide Names	Species Number
Amides/Anilines	Dimethazone, Oxadiazon, Procymidone, Acetochlor, Alachlor, Bentazon methyl *^d^*, Buprofezin, Butachlor, Cycluron, Dimethachlor, Fenoxanil, Mefenacet, Metalaxyl, Metazachlor, Metolachlor, Pretilachlor, s-Metolachlor	17
Azoles	Anvil, Epoxiconazole, Paclobutrazol, Propiconazole, Tebuconazole, Tricyclazole, Uniconazole	7
Carbamates	Baycarb, Isoprocarb, Pirimicarb, Propoxur	4
Heterocyclic	Fenson, Fludioxonil, Isoprothiolane, Picoxystrobin, Tebuthiuron	5
Organophosphates	Bolstar, Chlorpyrifos, Demeton, Diazinon, Dichlorvos, Disulfoton, Ethoprop, Fenthion, Malathion, Mevinphos, Phorate, Ronnel, Sulfotep, Tokuthion, Trichloronate	14
Triazines	Ametryn, Atrazine, Atrazine-desisopropyl *^d^*, Desethylatrazine *^d^*, Gesatamine, Metribuzin, Prebane, Prometon, Prometryn, Propazine, Sebuthylazin, Simazine, Simetryn	13

Note: *^d^* means pesticide’s degradation product.

**Table 2:** Correlation coefficient of the contaminants in drainage, Da Xingkai Lake and Xiao Xingkai Lake water.

	Vegetative Period			Maturity Period		
	Drainage Water	Da Xingkai Lake Water	Xiao Xingkai Lake Water	Drainage Water	Da Xingkai Lake Water	Xiao Xingkai Lake Water
Drainage water	1			1		
Da Xingkai Lake water	0.88242	1		0.8979	1	
Xiao Xingkai Lake water	0.86558	0.80537	1	0.89893	0.91036	1

**Table 3:** Acute aquatic life benchmarks of USEPA.

Pesticide	Year Updated	Freshwater Vertebrate (µg/L)	Freshwater Invertebrates (µg/L)
Atrazine	2016	2650	360
Chlorpyrifos	2022	0.85	0.0069
Prometryn	2014	1455	4850

## Data Availability

Data are contained within the article and supplementary materials. Other data will be made available on request.

## Supplementary Materials

The following supporting information can be downloaded at: https://www.mdpi.com/article/10.3390/toxics12010085/s1, Figure S1: Species sensitivity distribution (SSD) of 24 pesticides in Xingkai Lake water; Figure S2: The results of extraction solvent optimization; Figure S3 The results of elution solvent optimization; Table S1: Details of sampling sites; Table S2: Details, quantitative and qualitative ion (m/z), retention time, regression curve parameter, mean recoveries, the limit of detection (LOD), the limit of quantitation (LOQ) and supply company of 57 pesticides and three degradation products; Table S3: Summary of regression parameters and regression coefficient of species sensitivity distribution (SSD); Table S4: The mean concentrations of the 60 contaminants detected in sowing, vegetative and maturity periods in surrounding water (ng·L^-1^); Table S5: The mean concentrations of the 60 contaminants detected in dry and paddy field soil (ng·g^-1^); Table S6: Octanol-water partition coefficient of 57 pesticides and 3 degradations.
