# Supplementary material for: Distribution Characteristics and Risk Assessment of 57 Pesticides in Farmland Soil and the Surrounding Water
Source: Toxics. 2024 Jan 18;12(1):85. doi: 10.3390/toxics12010085 (PMC10818738; doi:10.3390/toxics12010085)
Supplement: Supplementary file 1 [file toxics-12-00085-s001.zip › toxics-2798927-supplementary.pdf]

Supplementary Materials: Distribution Characteristics and Risk Assessment of 57 Pesticides in Farmland Soil and the Surrounding Water

Weiqing Wang, Donghong Wang, Quanzhen Liu, Lihua Lin, Yongchang Xie, Chuan Du

Table S1 Details of sampling sites

Site	Position	Longitude E (°)	Latitude N (°)	Type of samples	Sampling period
D1	Xingkai Lake Farm drainage	132.875291	45.357833	Surface water	Sowing period, vegetative period and maturity period
D2	Xingkai Lake Farm main drainage	132.807865	45.288962	Surface water	Sowing period, vegetative period and maturity period
D3	Changlinzi drainage	132.307402	45.348883	Surface water	Sowing period, vegetative period and maturity period
D4	Chengzi river drainage	132.403317	45.454829	Surface water	Sowing period, vegetative period and maturity period
L1	Da Xingkai Lake	132.627942	45.298944	Surface water	Vegetative period and maturity period
L2	Da Xingkai Lake	132.188575	45.330419	Surface water	Vegetative period and maturity period
L3	Xiao Xingkai Lake	132.775032	45.263043	Surface water	Vegetative period and maturity period
L4	Xiao Xingkai Lake	132.581255	45.314456	Surface water	Vegetative period and maturity period
S1	Chengzihe Farm	132.291722	45.436892	Soybean base soil	Vegetative period
S2	Baiyuwan Farm	132.209365	45.413395	Soybean base soil	Maturity period
S3	Chengzihe Farm	132.363748	45.445875	Soybean base soil	Maturity period
S4	Xingkai Lake Farm	132.907559	45.328652	Soybean base soil	Maturity period
C1	Baiyuwan Farm	132.207321	45.334397	Corn base soil	Vegetative period
C2	Baiyuwan Farm	132.215229	45.419064	Corn base soil	Maturity period
C3	Xingkai Lake Farm	132.931234	45.312317	Corn base soil	Maturity period
P1	Xingkai Lake Farm paddy field	132.87876	45.366899	Surface water	Vegetative period
P2	Xingkai Lake Farm paddy field	132.807147	45.288541	Surface water	Sowing period and vegetative period
P3	Changlinzi Farm paddy field	132.284454	45.348337	Surface water	Sowing period and vegetative period
P4	Chengzihe Farm paddy field	132.403182	45.454134	Surface water	Sowing period and vegetative period
P1	Xingkai Lake Farm paddy field	132.87876	45.366899	Paddy base soil	Vegetative period
P2	Xingkai Lake Farm paddy field	132.807147	45.288541	Paddy base soil	Vegetative period
P3	Changlinzi Farm paddy field	132.284454	45.348337	Paddy base soil	Vegetative period
P4	Chengzihe Farm paddy field	132.403182	45.454134	Paddy base soil	Vegetative period
P5	Baiyuwan Farm paddy field	132.201172	45.365331	Paddy base soil	Maturity period
P6	Xingkai Lake Farm paddy field	132.799877	45.282703	Paddy base soil	Maturity period
P7	Xingkai Lake Farm paddy field	132.841185	45.277148	Paddy base soil	Maturity period

Table S2 Details, quantitative and qualitative ion (m/z), retention time, regression curve parameter, mean recoveries, the limit of detection (LOD), the limit of quantitation (LOQ) and supply company of 57 pesticides and three degradation products

Name	CAS	LOD ($ng·L^{-1}$)	LOQ ($ng·L^{-1}$)	Quantitative ion (m/z)	Qualitative ion1 (m/z)	Qualitative ion2 (m/z)	Retention time (min)	Linear term	Constant term	R^2	Mean recovery in water (%)	Mean recovery in soil (%)	Supply company
Acetochlor	34256-82-1	0.001	0.0025	146	162	132	15.301	3.108E+01	3.670E+02	0.9983	100.00	102.35	Accustandard
Alachlor	15972-60-8	0.005	0.01	160	188	237	17.378	4.166E+01	1.157E+02	0.9998	97.82	83.92	Accustandard
Ametryn	834-12-8	0.0025	0.01	212	227	170	17.839	4.773E+01	1.988E+02	0.9996	96.49	89.04	Accustandard
Anvil	79983-71-4	0.005	0.025	214	83	216	20.406	3.553E+01	3.315E+02	0.9998	101.70	93.01	Anpel
Atrazine	1912-24-9	0.001	0.0025	200	215	202	13.372	5.339E+01	5.376E+02	0.999	98.80	109.32	Accustandard
Atrazine-desisopropyl	1007-28-9	0.005	0.025	158	173	145	11.686	1.892E+01	6.537E+01	0.9996	81.70	86.87	Accustandard
Baycarb	3766-81-2	0.0025	0.01	121	122	150	9.291	9.908E+01	1.691E+02	0.9996	91.76	93.70	Accustandard
Bentazon methyl	61592-45-8	0.0025	0.01	212	105	104	15.986	6.550E+01	5.464E+02	0.9992	99.62	95.31	Anpel
Bolstar	35400-43-2	0.01	0.025	322	156	140	25.485	4.940E+01	-2.424E+02	0.9993	80.64	69.69	Accustandard
Buprofezin	69327-76-0	0.01	0.025	172	175	305	25.861	2.221E+01	-3.623E+00	0.9998	97.07	92.55	Accustandard
Butachlor	23184-66-9	0.001	0.0025	176	160	188	19.213	5.474E+01	5.706E+02	0.999	102.90	88.23	Accustandard
Chlorpyrifos	2921-88-2	0.001	0.0025	314	316	197	16.902	3.697E+01	4.335E+02	0.9985	104.30	96.46	Accustandard
Cycluron	8015-55-2	0.005	0.01	72	89	127	13.631	2.907E+01	3.787E+02	0.9992	101.10	88.88	Accustandard
Demeton	8065-48-3	0.01	0.025	88	89	171	12.635	5.419E+00	2.823E+01	0.9993	74.81	88.27	Accustandard
Desethylatrazine	6190-65-4	0.0025	0.005	172	187	145	11.869	6.458E+01	4.657E+02	0.999	96.00	86.35	Accustandard
Diazinon	333-41-5	0.005	0.01	304	137	179	15.823	6.963E+00	-2.740E+01	0.9993	94.45	100.51	Accustandard
Dichlorvos	62-73-7	0.0025	0.005	185	187	220	8.066	2.618E+01	1.413E+02	0.9993	97.78	96.80	Accustandard
Dimethachlor	50563-36-5	0.0025	0.01	134	197	132	16.933	1.176E+02	5.161E+02	0.9997	97.95	74.10	Accustandard
Dimethazone	81777-89-1	0.001	0.0025	204	125	127	13.302	6.580E+01	4.657E+02	0.999	97.50	98.36	Accustandard
Epoxiconazole	133855-98-8	0.005	0.01	111	138	165	23.477	1.553E+01	4.015E+02	0.9989	87.70	85.04	TMRM
Ethoprop	13194-48-4	0.01	0.025	158	200	242	13.043	4.476E+00	2.075E+01	0.9994	91.00	65.06	Accustandard
Fenoxanil	115852-48-7	0.0025	0.01	189	191	293	21.396	1.373E+02	1.245E+03	0.9992	95.59	96.10	Anpel
Fenson	80-38-6	0.0025	0.01	141	77	268	18.024	6.831E+01	4.232E+02	0.9992	102.04	96.65	Anpel
Fenthion	55-38-9	0.005	0.01	278	153	169	19.728	4.581E+01	-3.023E+02	0.999	77.72	80.77	Accustandard
Fludioxonil	131341-86-1	0.005	0.025	248	154	127	25.934	5.213E+01	-2.730E+01	0.9999	91.92	98.80	Accustandard
Gesatamine	1610-17-9	0.0025	0.01	196	211	169	13.477	8.073E+01	7.340E+02	0.9993	100.43	94.26	Accustandard
Isoprocarb	2631-40-5	0.0025	0.01	121	136	103	8.057	7.437E+01	-1.988E+02	0.9999	90.09	89.34	Accustandard
Isoprothiolane	50512-35-1	0.001	0.0025	118	162	189	19.896	4.386E+01	4.028E+02	0.9992	101.40	103.35	Accustandard
Malathion	121-75-5	0.025	0.05	173	158	143	19.035	7.825E+00	-2.398E+01	0.9998	100.68	65.07	Accustandard
Mefenacet	73250-68-7	0.0025	0.005	192	136	120	25.562	1.117E+02	2.350E+03	0.9986	86.10	97.88	Accustandard
Metalaxyl	57837-19-1	0.0025	0.01	206	132	160	16.179	3.410E+01	2.946E+02	0.9991	100.83	92.90	Rhawn
Metazachlor	67129-08-2	0.01	0.05	132	133	209	20.071	2.717E+01	7.089E+01	0.9997	100.01	91.42	Accustandard

Metolachlor	51218-45-2	0.0025	0.01	238	162	240	17.194	5.142E+01	1.886E+02	0.9997	100.15	95.06	Accustandard
Metribuzin	21087-64-9	0.0025	0.01	198	199	103	15.826	1.535E+01	2.296E+02	0.9992	99.36	99.00	Anpel
Mevinphos	7786-34-7	0.01	0.025	127	192	164	9.643	5.749E+00	-1.658E+01	0.9993	98.14	73.00	Accustandard
Oxadiazon	19666-30-9	0.001	0.0025	175	258	177	20.624	4.825E+02	2.663E+03	0.9993	101.77	99.35	Anpel
Paclobutrazol	76738-62-0	0.0025	0.005	236	125	167	19.278	7.063E+01	4.318E+02	0.9994	98.30	84.88	Accustandard
Phorate	298-02-2	0.01	0.025	260	121	231	14.205	6.963E+00	-2.740E+01	0.9993	90.24	69.31	Accustandard
Picoxystrobin	117428-22-5	0.005	0.025	335	146	145	24.446	4.542E+01	-1.255E+02	0.9999	93.46	88.60	Accustandard
Pirimicarb	23103-98-2	0.0025	0.01	166	72	238	17.981	1.032E+02	1.076E+02	0.9999	90.75	64.30	Accustandard
Prebane	886-50-0	0.005	0.01	226	185	241	20.575	7.662E+01	-7.838E+01	0.9999	98.04	85.91	Accustandard
Pretilachlor	51218-49-6	0.005	0.01	202	238	262	20.297	1.157E+01	-3.329E+01	0.9998	100.31	97.56	Rhawn
Procymidone	32809-16-8	0.005	0.025	283	285	284	23.631	4.732E+01	-2.318E+02	0.9997	93.90	68.70	Accustandard
Prometon	1610-18-0	0.005	0.01	210	225	168	14.979	3.257E+01	1.997E+02	0.9996	98.39	94.47	Accustandard
Prometryn	7287-19-6	0.001	0.0025	241	226	184	16.102	6.275E+01	6.769E+02	0.9984	100.20	93.65	Accustandard
Propazine	139-40-2	0.005	0.01	214	216	229	15.188	5.569E+01	2.581E+02	0.9991	97.70	91.29	Accustandard
Propiconazole	60207-90-1	0.005	0.01	259	261	173	22.391	3.046E+01	6.310E+01	0.9993	95.60	81.83	Accustandard
Propoxur	114-26-1	0.005	0.01	110	152	111	7.488	1.100E+02	-1.545E+02	0.9999	92.99	60.88	Accustandard
Ronnel	299-84-3	0.005	0.01	285	287	125	18.448	3.344E+01	-1.984E+02	0.9991	97.59	90.04	Accustandard
Sebuthylazin	7286-69-3	0.0025	0.01	200	229	202	16.556	1.365E+02	4.231E+00	0.9999	97.92	88.47	Accustandard
Simazine	122-34-9	0.01	0.025	201	202	186	14.954	1.047E+01	1.203E+02	0.9993	98.06	91.70	Accustandard
Simetryn	1014-70-6	0.001	0.0025	170	198	213	15.875	8.247E+01	7.90E+02	0.9989	100.70	94.92	Accustandard
s-Metolachlor	87392-12-9	0.0025	0.01	162	238	240	18.695	1.235E+02	6.034E+02	0.9995	98.30	95.79	Accustandard
Sulfotep	3689-24-5	0.001	0.0025	322	202	238	13.608	7.840E+01	-2.197E+02	0.9998	91.84	60.98	Accustandard
Tebuconazole	107534-96-3	0.005	0.01	250	252	125	23.133	4.842E+01	6.773E+01	0.9997	93.00	87.18	Accustandard
Tebuthiuron	34014-18-1	0.01	0.025	156	171	74	12.625	5.749E+01	-6.519E+02	0.9996	97.90	85.40	Accustandard
Tokuthion	34643-46-4	0.01	0.025	309	267	162	23.088	2.189E+01	-1.839E+02	0.9992	98.08	75.56	Accustandard
Trichloronate	327-98-0	0.005	0.01	297	269	109	20.229	4.123E+01	5.290E+01	0.9996	100.69	67.97	Accustandard
Tricyclazole	41814-78-2	0.0025	0.005	162	189	161	20.041	3.378E+01	-5.741E+02	0.9993	89.90	89.80	Accustandard
Uniconazole	83657-22-1	0.005	0.025	234	70	236	20.843	5.144E+01	1.682E+02	0.9993	89.80	89.58	Anpel

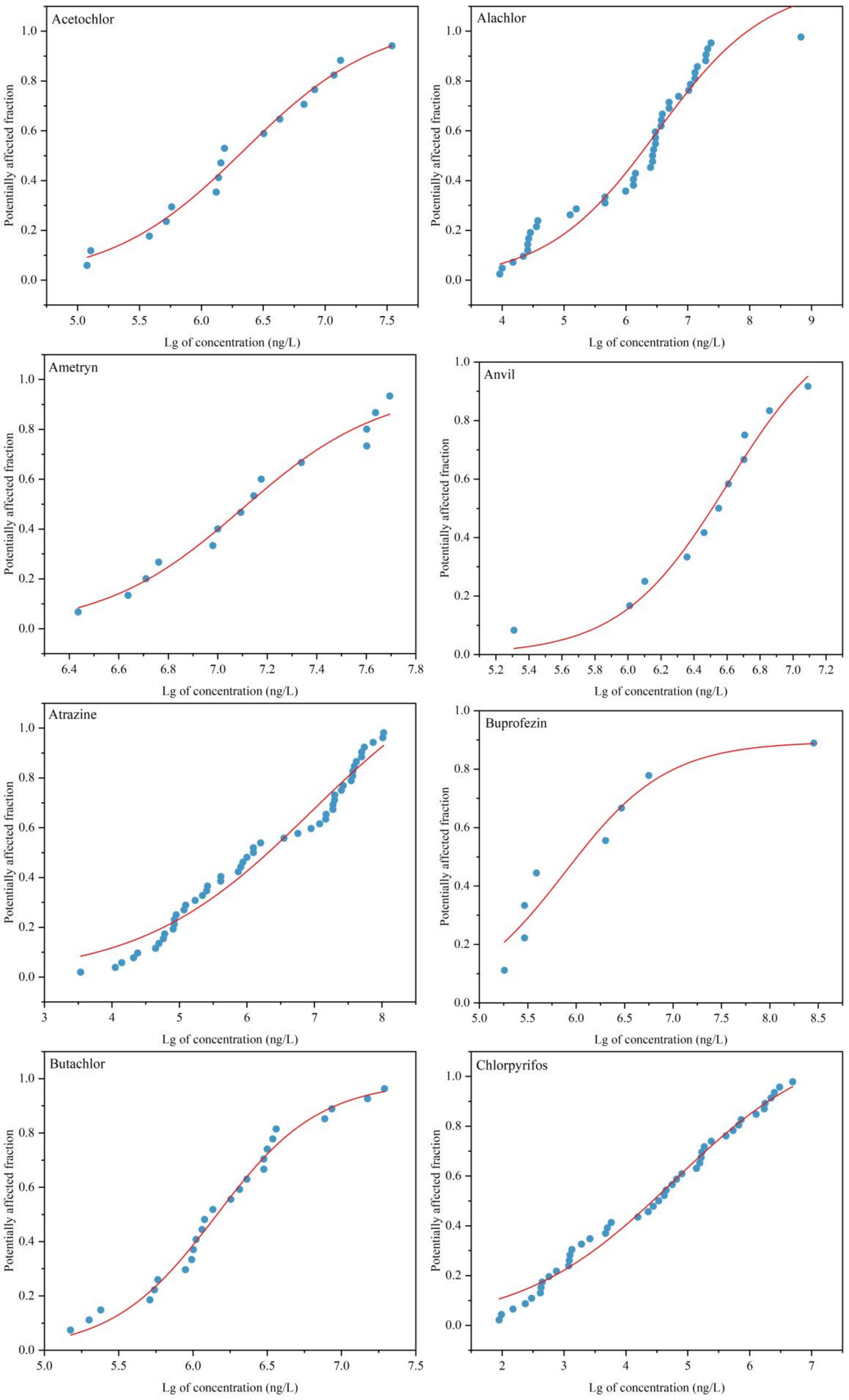

Acetochlor
Alachlor
Ametryn
Anvil
Atrazine
Buprofezin
Butachlor
Chlorpyrifos
Potentially affected fraction
Lg of concentration (ng/L)

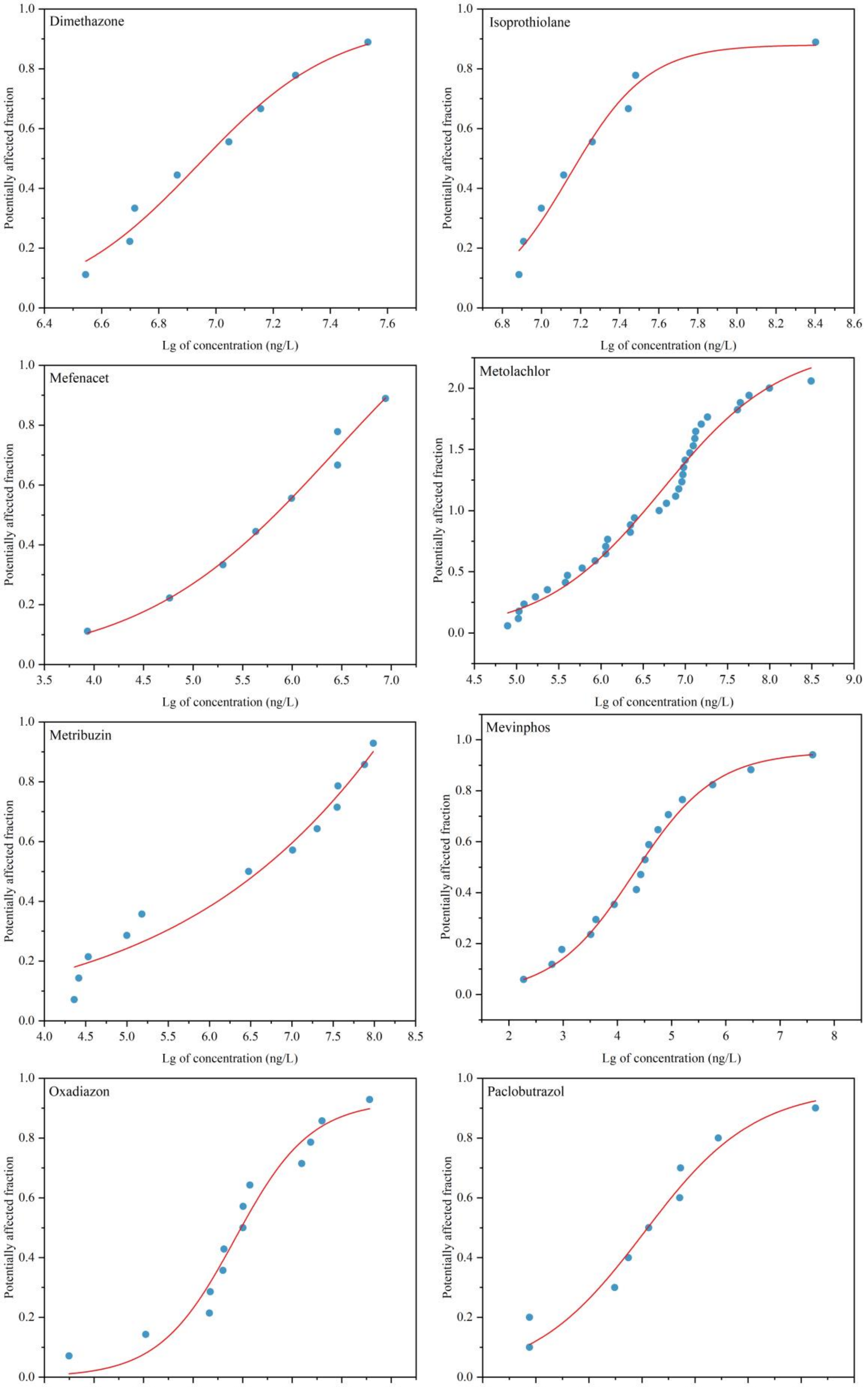
Dimethazone
Isoprothiolane
Mefenacet
Metolachlor
Metribuzin
Mevinphos
Oxadiazon
Paclobutrazol
Potentially affected fraction
Lg of concentration (ng/L)

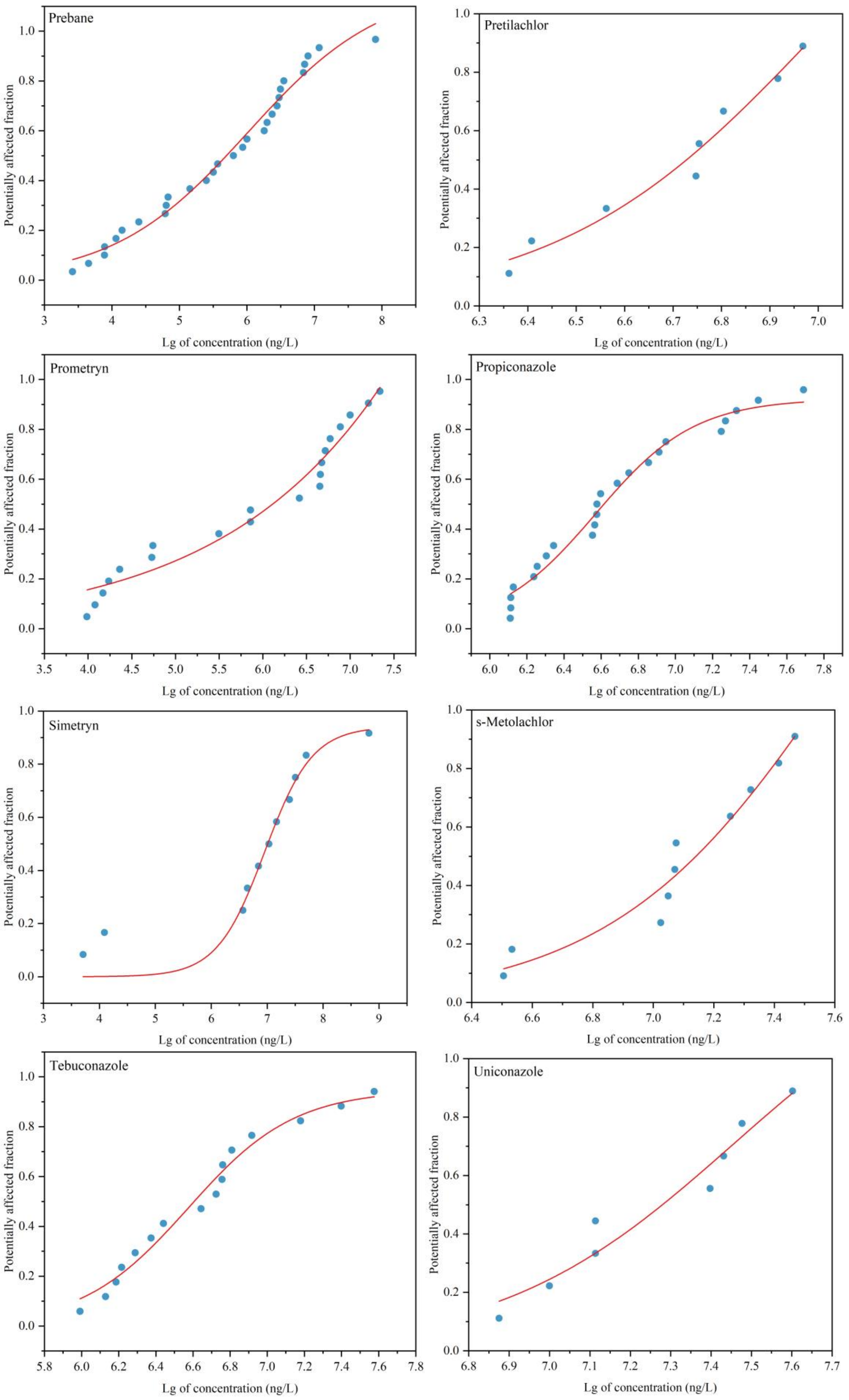

Fig. S1 Species sensitivity distribution (SSD) of 24 pesticides in Xingkai Lake water

Table S3 Summary of regression parameters and regression coefficient of species sensitivity distribution (SSD)

Name	a	x_c	k	r^2
Acetochlor	1.04465	6.34559	1.84754	0.98163
Alachlor	1.19355	6.50733	1.12478	0.95374
Ametryn	0.96607	7.10083	3.53568	0.97272
Anvil	1.15953	6.60032	3.0931	0.97593
Atrazine	1.32864	6.95324	0.79085	0.96812
Buprofezin	0.89368	5.88436	1.90421	0.93377
Butachlor	0.98903	6.15377	2.87509	0.98575
Chlorpyrifos	1.15908	4.77123	0.81431	0.98485
Dimethazone	0.95704	6.9376	4.15432	0.98138
Isoprothiolane	0.88012	7.14114	5.01159	0.97295
Mefenacet	1.42393	6.43445	1.01052	0.98778
Metolachlor	2.34553	6.72841	1.41239	0.97579
Metribuzin	5.91165	11.55488	0.48092	0.96708
Mevinphos	0.95299	4.30979	1.33809	0.98674
Oxadiazon	0.92348	6.42096	2.6151	0.95738
Paclobutrazol	0.9661	7.30349	9.56373	0.9685
Prebane	1.18501	6.01131	1.00055	0.98309
Pretilachlor	1.84605	6.9915	3.75588	0.97128
Prometryn	21.71838	12.85222	0.5661	0.9609
Propiconazole	0.92278	6.57083	3.75252	0.97709
Simetryn	0.94119	6.95528	2.34654	0.95058
s-Metolachlor	2.33626	7.64072	2.60514	0.95078
Tebuconazole	0.94742	6.57456	3.49579	0.97865
Uniconazole	1.41694	7.4565	3.43266	0.95191

Accelerated solvent extraction (ASE) condition optimization

(1) Optimization of extraction solvent

A total of three groups of experiments with different extraction solvents were set up, and three parallel experiments were set up for each group. The extraction solvents were n-hexane (HEX): acetone (ACE) (1: 1, v/v), HEX: ACE: dichloromethane (DCM) (1: 1: 1, v/v/v), and DCM: ACE (1: 1, v/v). The rest of the soil pretreatment methods were the same, and the specific methods were as follows:

The soil was sieved to a particle size of less than 2 mm after being freeze-dried using Freeze Dry Systems (Freezone 4.5, Labconco, USA). Accelerated solvent extraction (ASE) was carried out with an ASE 350 extractor (Dionex, USA). The extraction program for soil samples was as follows: 5 g of soil was mixed with 2 g of celite in a 34 mL stainless steel vessel, and 150 ng of target compounds was added; heating at 100 °C, 1500 psi for 5 min, static extraction for 5 min, and cycling for two times; and the extraction cell was flushed with 60% of the cell volume of the solvent and purged with nitrogen for 60 s. The extract was dehydrated using anhydrous sodium sulfate and then concentrated to 2 mL using a rotary evaporator (Heidolph, Germany). After the extraction procedures, the extracts were transferred to a Florisil column, which was used to clean up interfering substances. The columns were activated with 10 mL each of HEX and DCM before use. The target components were eluted with 15 mL of HEX. The elute was concentrated to dryness with a gentle nitrogen flow, then the solvent was replaced with HEX, setting the volume to 0.5 mL, and maintained at −20 °C for subsequent instrumental analysis. The result is shown in Fig. S2. The extraction efficiency of the three extraction solvents were not obvious, so we chose HEX: ACE (1: 1, v/v) as the final extraction solvent.

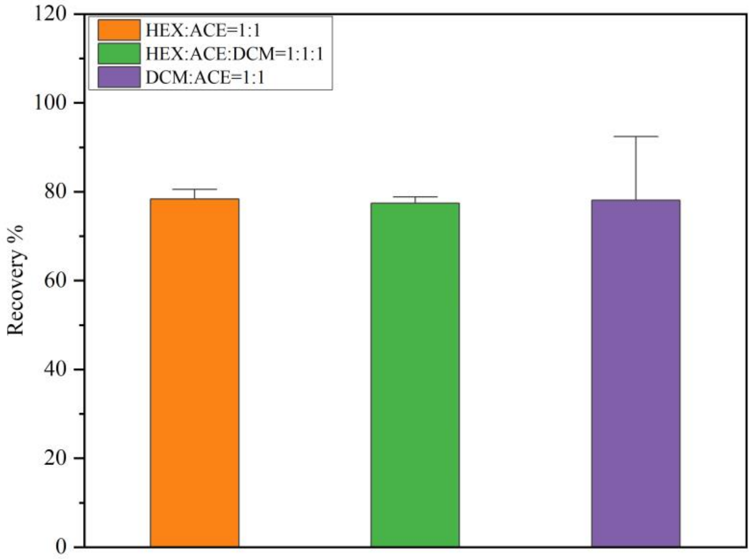

Fig. S2 The results of extraction solvent optimization

(2) Optimization of elution solvent

A total of two groups of experiments with different elution solvents were set up, and three parallel experiments were set up for each group. The elution solvents were HEX and HEX: DCM (1: 1, v/v). The rest of the soil pretreatment methods were the same, and the specific methods were as follows:

The soil was sieved to a particle size of less than 2 mm after being freeze-dried using Freeze Dry Systems (Freezone 4.5, Labconco, USA). Accelerated solvent extraction (ASE) was carried out with an ASE 350 extractor (Dionex, USA). The extraction program for soil samples was as follows: 5 g of soil was mixed with 2 g of celite in a 34 mL stainless steel vessel, and 150 ng of target compounds was added; then, acetone: HEX (1: 1, v/v) was used as the extraction solvent at 100 ℃ under a pressure of 1500 psi with 5 min of heating and 5 min of static extraction, which was carried out in two cycles; and the extraction cell was flushed with 60% of the cell volume of the solvent and purged with nitrogen for 60 s. The extract was dehydrated using anhydrous sodium sulfate and then concentrated to 2 mL using a rotary evaporator (Heidolph, Germany). After the extraction procedures, the extracts were transferred to a Florisil column, which was used to clean up interfering substances. The columns were activated with 10 mL each of DCM and HEX before use. The target components were eluted with 15 mL of the elution solvent. The elute was concentrated to dryness with a gentle nitrogen flow, then the solvent was replaced with HEX, setting the volume to 0.5 mL, and maintained at −20 °C for subsequent instrumental analysis. The result is shown in Fig. S3. The recovery of HEX: DCM (1: 1, v/v) was better than that of HEX, so HEX: DCM (1: 1, v/v) was chosen as the elution solvent.

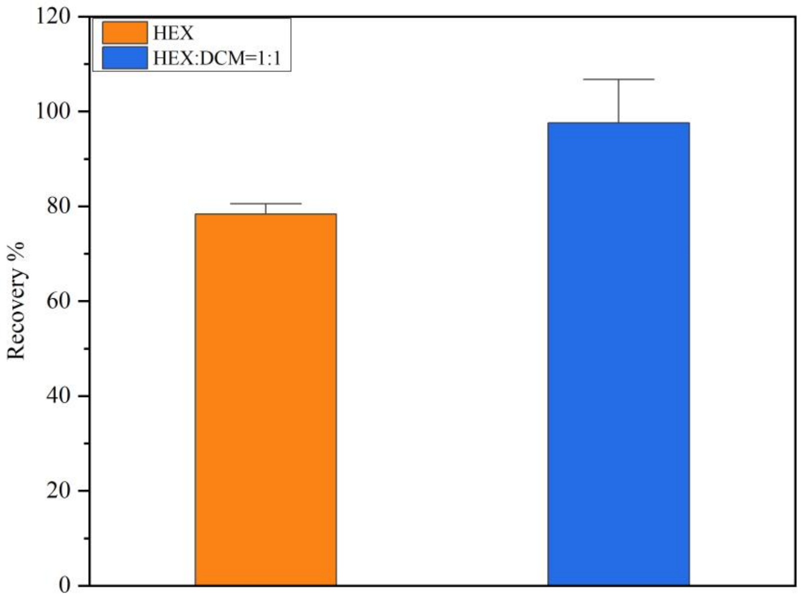

Fig. S3 The results of elution solvent optimization

Table S4 The mean concentrations of the 60 contaminants detected in sowing, vegetative and maturity periods in surrounding water (ng·L^{-1})

Name	Sowing period		Vegetative period				Maturity period		
	Paddy field	Drainage	Paddy field	Drainage	Da Xingkai Lake	Xiao Xingkai Lake	Drainage	Da Xingkai Lake	Xiao Xingkai Lake
Acetochlor	22.37	185.35	33.74	135.24	163.50	52.46	25.73	34.96	32.60
Alachlor	9.10	3.10	18.51	1.93	9.81	1.79	n.d.	n.d.	n.d.
Ametryn	1.78	2.50	13.90	9.36	1.83	3.94	0.62	4.04	1.58
Anvil	22.03	6.72	10.51	30.88	106.73	1.29	13.71	7.86	8.84
Atrazine	9.58	45.34	87.30	431.12	426.53	114.44	123.32	153.63	194.78
Atrazine-desisopropyl	0.48	0.57	1.28	24.87	19.45	6.11	2.60	10.52	8.55
Baycarb	3.66	2.47	6.74	n.d.	n.d.	n.d.	4.90	n.d.	7.67
Bentazon methyl	10.18	3.56	126.32	32.38	12.48	1.12	2.57	1.45	1.44
Bolstar	n.d.	n.d.	n.d.	n.d.	n.d.	n.d.	n.d.	n.d.	n.d.
Buprofezin	n.d.	n.d.	1498.25	498.66	501.72	243.76	201.38	181.69	280.31
Butachlor	8.14	20.62	143.30	36.10	108.51	16.71	7.38	9.88	11.23
Chlorpyrifos	0.43	1.38	47.56	14.01	14.86	0.68	0.40	n.d.	n.d.
Cycluron	n.d.	n.d.	9.77	n.d.	n.d.	n.d.	n.d.	n.d.	n.d.
Demeton	n.d.	n.d.	20.49	n.d.	8.37	15.50	14.34	19.32	4.77
Desethylatrazine	2.05	39.17	31.88	106.29	71.69	33.47	30.09	38.09	42.73
Diazinon	n.d.	n.d.	n.d.	n.d.	n.d.	n.d.	n.d.	n.d.	n.d.
Dichlorvos	2.05	n.d.	n.d.	n.d.	n.d.	n.d.	n.d.	n.d.	n.d.
Dimethachlor	2.28	n.d.	n.d.	n.d.	0.66	n.d.	n.d.	n.d.	n.d.
Dimethazone	5.27	68.53	40.56	124.43	64.08	29.46	17.05	29.06	39.29
Epoxiconazole	0.50	n.d.	n.d.	1.59	n.d.	n.d.	3.64	n.d.	n.d.
Ethoprop	n.d.	n.d.	n.d.	n.d.	n.d.	n.d.	n.d.	n.d.	n.d.
Fenoxanil	4.45	5.56	36.41	22.36	9.45	7.91	9.06	15.09	17.26
Fenson	1.91	2.79	4.05	18.15	n.d.	1.08	n.d.	n.d.	n.d.
Fenthion	n.d.	n.d.	11.60	2.03	1.87	n.d.	n.d.	n.d.	n.d.
Fludioxonil	n.d.	n.d.	n.d.	n.d.	n.d.	n.d.	n.d.	n.d.	n.d.
Gesatamine	n.d.	n.d.	23.44	59.84	112.37	17.54	6.59	13.26	11.40

Isoprocarb	1.94	n.d.	n.d.	n.d.	n.d.	n.d.	n.d.	n.d.	n.d.
Isoprothiolane	25.96	40.67	29.45	69.60	236.21	23.10	116.75	142.59	150.17
Malathion	2.66	1.58	59.13	n.d.	12.81	6.67	1.29	n.d.	n.d.
Mefenacet	4.24	n.d.	370.82	64.32	54.62	3.47	3.26	0.83	0.40
Metalaxyl	11.37	13.79	34.58	31.67	39.54	17.91	3.90	11.01	15.15
Metazachlor	n.d.	n.d.	2.55	42.69	15.75	n.d.	n.d.	n.d.	n.d.
Metolachlor	8.18	50.84	15.69	132.54	46.93	34.02	52.20	44.17	43.68
Metribuzin	n.d.	3.01	86.68	44.01	15.87	2.41	1.84	n.d.	n.d.
Mevinphos	n.d.	n.d.	70.77	62.21	36.73	47.04	25.57	30.72	27.57
Oxadiazon	42.78	2.12	98.51	28.71	24.11	2.62	5.55	1.19	1.74
Paclobutrazol	54.00	15.21	730.66	136.39	144.84	23.70	8.35	5.47	11.05
Phorate	n.d.	n.d.	n.d.	n.d.	n.d.	n.d.	n.d.	n.d.	n.d.
Picoxystrobin	n.d.	n.d.	n.d.	n.d.	n.d.	n.d.	n.d.	n.d.	n.d.
Pirimicarb	n.d.	n.d.	42.88	4.70	7.02	3.47	11.90	0.81	29.14
Prebane	n.d.	n.d.	38.90	68.89	84.88	28.58	9.38	22.29	n.d.
Pretilachlor	2.59	1.41	17.53	18.22	10.09	2.26	10.40	0.69	0.28
Procymidone	n.d.	n.d.	n.d.	n.d.	n.d.	n.d.	n.d.	n.d.	n.d.
Prometon	n.d.	1.98	n.d.	19.79	n.d.	n.d.	3.21	n.d.	5.76
Prometryn	15.69	89.44	70.37	80.00	78.66	25.49	21.85	28.20	36.80
Propazine	n.d.	n.d.	5.23	6.26	n.d.	3.23	0.41	n.d.	n.d.
Propiconazole	2.88	2.71	9.40	25.83	109.28	1.03	2.93	1.98	2.24
Propoxur	1.40	n.d.	n.d.	n.d.	n.d.	n.d.	n.d.	n.d.	n.d.
Ronnel	n.d.	n.d.	n.d.	n.d.	n.d.	n.d.	n.d.	n.d.	n.d.
Sebuthylazin	n.d.	n.d.	n.d.	n.d.	n.d.	n.d.	n.d.	n.d.	n.d.
Simazine	2.32	28.01	2.29	n.d.	n.d.	n.d.	n.d.	n.d.	n.d.
Simetryn	20.10	19.43	490.47	164.96	292.00	51.53	22.06	41.81	65.71
s-Metolachlor	n.d.	n.d.	37.67	20.85	1.47	15.73	4.02	n.d.	n.d.
Sulfotep	n.d.	n.d.	0.64	0.44	n.d.	n.d.	n.d.	n.d.	n.d.
Tebuconazole	16.87	9.39	85.35	49.19	77.00	41.95	30.98	17.63	21.01

Tebuthiuron	n.d.	n.d.	5.76	9.70	n.d.	n.d.	5.22	3.14	2.57
Tokuthion	n.d.	n.d.	n.d.	6.48	n.d.	4.33	n.d.	n.d.	n.d.
Trichloronate	13.03	n.d.	n.d.	n.d.	11.59	n.d.	n.d.	n.d.	n.d.
Tricyclazole	9.67	40.78	68.82	53.51	46.68	65.17	33.68	41.68	57.12
Uniconazole	4.55	9.05	174.17	1.17	n.d.	0.84	3.63	1.50	0.84

Note: n.d. means the result is lower than the detection limit.

Table S5 The mean concentrations of the 60 contaminants detected in dry and paddy field soil ($ng \cdot g^{-1}$)

Name	Sowing period		Vegetative period	
	Dry field	Paddy field	Dry field	Paddy field
Acetochlor	80.49	10.83	36.50	12.30
Alachlor	1.61	1.22	0.28	0.66
Ametryn	n.d.	3.11	n.d.	0.30
Anvil	n.d.	0.20	n.d.	6.79
Atrazine	67.48	0.09	9.53	0.19
Atrazine-desisopropyl	2.83	1.92	1.18	0.54
Baycarb	0.43	2.51	0.40	n.d.
Bentazon methyl	0.64	5.81	n.d.	4.28
Bolstar	n.d.	0.26	0.39	n.d.
Buprofezin	n.d.	n.d.	n.d.	4.03
Butachlor	1.59	8.46	0.15	10.96
Chlorpyrifos	101.21	16.54	n.d.	24.69
Cycluron	32.93	21.51	5.89	4.76
Demeton	n.d.	2.98	0.42	0.98
Desethylatrazine	6.08	n.d.	0.59	n.d.
Diazinon	n.d.	n.d.	n.d.	n.d.
Dichlorvos	n.d.	n.d.	n.d.	n.d.
Dimethachlor	1.93	1.21	1.87	0.09
Dimethazone	355.52	4.01	13.64	6.68
Epoxiconazole	n.d.	n.d.	n.d.	n.d.
Ethoprop	n.d.	4.57	0.74	1.55
Fenoxanil	n.d.	n.d.	n.d.	0.95
Fenson	1.28	2.66	0.43	1.84
Fenthion	12.04	2.00	n.d.	2.99
Fludioxonil	n.d.	n.d.	n.d.	n.d.
Gesatamine	n.d.	0.19	n.d.	n.d.
Isoprocarb	n.d.	0.14	n.d.	n.d.
Isoprothiolane	2.18	3.77	n.d.	3.90
Malathion	3.51	2.87	1.62	5.62
Mefenacet	44.37	119.84	n.d.	23.62
Metalaxyl	0.77	1.40	0.82	1.04
Metazachlor	n.d.	n.d.	n.d.	n.d.
Metolachlor	0.48	n.d.	4.20	n.d.
Metribuzin	n.d.	n.d.	n.d.	n.d.
Mevinphos	6.85	24.07	10.31	12.97
Oxadiazon	10.88	15.81	n.d.	12.34
Paclobutrazol	0.73	6.04	0.17	1.57
Phorate	n.d.	n.d.	n.d.	n.d.
Picoxystrobin	n.d.	n.d.	n.d.	n.d.
Pirimicarb	2.41	6.04	0.51	5.33
Prebane	0.90	0.63	n.d.	n.d.
Pretilachlor	n.d.	7.27	n.d.	5.05
Procymidone	n.d.	1.46	n.d.	0.55
Prometon	0.76	1.63	3.97	7.31
Prometryn	4.38	1.17	0.38	6.01

Propazine	n.d.	n.d.	n.d.	n.d.
Propiconazole	0.56	4.80	0.60	2.35
Propoxur	n.d.	n.d.	n.d.	n.d.
Ronnel	n.d.	n.d.	n.d.	n.d.
Sebuthylazin	n.d.	n.d.	n.d.	n.d.
Simazine	7.91	n.d.	0.04	0.27
Simetryn	2.47	14.77	n.d.	2.22
s-Metolachlor	2.21	0.90	7.66	0.19
Sulfotep	n.d.	n.d.	n.d.	n.d.
Tebuconazole	n.d.	5.00	0.60	16.76
Tebuthiuron	0.70	0.79	n.d.	n.d.
Tokuthion	0.84	n.d.	1.30	n.d.
Trichloronate	n.d.	n.d.	n.d.	n.d.
Tricyclazole	1.66	5.32	4.06	6.36
Uniconazole	n.d.	n.d.	n.d.	n.d.

Note: n.d. means the result is lower than the detection limit.

Table S6 Octanol-water partition coefficient of 57 pesticides and 3 degradations

Name	Octanol-water partition coefficient (Kow) at pH 7, 20 °C<br>LogKow
Acetochlor	4.14
Alachlor	3.09
Ametryn	2.63
Anvil	3.9
Atrazine	2.7
Atrazine-desisopropyl	1.15
Baycarb	2.78
Bentazon methyl	-
Bolstar	5.48
Buprofezin	4.5
Butachlor	4.5
Chlorpyrifos	4.7
Cycluron	2.84
Demeton	3.21
Desethylatrazine	1.51
Diazinon	3.69
Dichlorvos	1.9
dimethachlor	2.17
Dimethazone	2.58
Epoxiconazole	3.3
Ethoprop	2.99
Fenoxanil	3.35
Fenson	3.57
Fenthion	4.84
Fludioxonil	4.12
Gesatamine	2.69
Isoprocarb	2.32
Isoprothiolane	3.3
Malathion	2.75
Mefenacet	3.23
Metalaxyl	1.75
Metazachlor	2.49
Metolachlor	3.4
Metribuzin	1.7
Mevinphos	0.127
Oxadiazon	5.33
Paclobutrazol	3.11
Phorate	3.86
Picoxystrobin	3.6
Pirimicarb	1.7
Prebane	3.66
Pretilachlor	4.08
Procymidone	3.3
prometon	2.91
Prometryn	3.34
Propazine	3.95

Propiconazole	3.72
Propoxur	0.14
Ronnel	4.88
Sebuthylazin	-
Simazine	2.3
Simetryn	2.8
s-Metolachlor	3.05
Sulfotep	3.99
Tebuconazole	3.7
Tebuthiuron	1.79
Tokuthion	-
Trichloronate	5.23
Tricyclazole	1.4
Uniconazole	3.84

Note: Data from PPDB, Pesticides Properties Data Base, and University of Hertfordshire; “-” means the data were not provided.
